# Lowering pain and inflammation drug costs: evaluating the impact of a cost plus drug company model

**DOI:** 10.1093/pm/pnaf034

**Published:** 2025-03-26

**Authors:** Kaylyn Rowsey, Adam Khan, Jillian Brassfield, Matthew Rashid, Jacob Duncan, Matt Vassar

**Affiliations:** Office of Medical Student Research, Oklahoma State University Center for Health Sciences, Tulsa, OK 74107, United States; Office of Medical Student Research, Oklahoma State University Center for Health Sciences, Tulsa, OK 74107, United States; Office of Medical Student Research, Oklahoma State University Center for Health Sciences, Tulsa, OK 74107, United States; Office of Medical Student Research, Oklahoma State University Center for Health Sciences, Tulsa, OK 74107, United States; Office of Medical Student Research, Oklahoma State University Center for Health Sciences, Tulsa, OK 74107, United States; Office of Medical Student Research, Oklahoma State University Center for Health Sciences, Tulsa, OK 74107, United States; Department of Psychiatry and Behavioral Sciences, Oklahoma State University Center for Health Sciences, Tulsa, OK 74107, United States

Dear Editor,

## Introduction

In 2022, Americans spent $405.9 billion on prescription medications, driven largely by rising drug prices rather than increased demand, intensifying the financial burden on consumers.^1^ That year, Medicare spending reached $944.3 billion (21% of national health expenditures), and by July 2024, enrollment had grown to 67.7 million, with more than 80% of beneficiaries receiving Part D coverage.^1^^,^^2^ With the elderly projected to comprise 25% of the US population by 2060, sustainable health care solutions are needed.^3^ Rising Medicare Part D costs, driven by inflation and limited price negotiation, add strain, with a 2023 survey showing one third of Medicare beneficiaries delaying care because of costs, 78% of whom suffer from chronic pain. (https://tinyurl.com/2raxdsy4).^4^

The Mark Cuban Cost Plus Drug Company (MCCPDC) offers an innovative solution by providing prescription medications at reduced prices through a transparent pricing model. By bypassing intermediaries such as pharmacy benefit managers (PBMs), MCCPDC ensures that patients pay closer to the actual cost of medications.^5^ Our study evaluates how MCCPDC’s pricing compares with Medicare Part D rates and its potential to alleviate costs of pain and inflammation medications, contributing to the program's long-term sustainability.

## Methods

### Study design

We conducted a cross-sectional analysis comparing MCCPDC prices with the most recent publicly available Medicare Part D spending data (2022). The protocol followed CHEERS guidelines and is available on the Open Science Framework (OSF) (https://osf.io/93g7f/).

### Definitions

- “Count” indicates the number of pills or doses within a prescription. For example, a “30-count” prescription includes 30 pills, while a “90-count” prescription has 90 pills, signifying the total pill quantity in that prescription.
- “Unit” represents cost per pill. When we refer to “unit cost,” we mean the price of a single pill.

### Inclusion/exclusion criteria and data extraction

We compared “Pain & Inflammation” drugs listed by MCCPDC with 2022 Medicare Part D data, excluding those not matched or without 30- or 90-count options. We used the highest-cost dosage for conservative estimates. Two authors independently extracted data, resolving discrepancies by consensus. Our analysis included exclusively generic medications, consistent with MCCPDC's current drug inventory.

### Data analysis

To estimate potential savings, we compared Medicare and MCCPDC unit prices for 30- and 90-count prescriptions. Medicare Part D spending data obtained (https://tinyurl.com/5fmxyk77) reports gross drug costs, including Medicare reimbursements, plan-paid amounts, and beneficiaries’ out-of-pocket payments (coinsurance, copayments). Pharmacy acquisition costs, rebates, and other confidential price concessions are not itemized separately in these data, as they are encompassed within the total reported expenditures. Our analysis, therefore, reflects these combined payments to provide a comprehensive economic assessment. For conservative estimates, we added a $5 shipping fee to the highest-cost items and multiplied MCCPDC unit prices by 2022 volume-adjusted quantities. Given that Centers for Medicare & Medicaid Services (CMS) does not separately report pharmacy acquisition costs or manufacturer rebates, our estimates reflect combined Medicare and beneficiary spending rather than net payer-specific savings. Calculations are available on OSF (https://osf.io/93g7f/).

## Results

**Figure 1.**
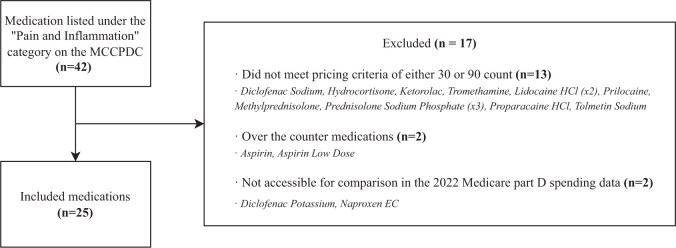
Flow diagram for medications inclusion.

We analyzed 25 medications, excluding drugs that did not meet pricing criteria or were unavailable in the 2022 Medicare Part D data (Figure 1). Medicare spent $690 million on these drugs, and if contracted rates had matched MCCPDC’s 2024 prices, potential savings would total $344 million (Table 1). For 30-count prescriptions, 19 of 25 drugs (76%) showed potential savings, totaling $200 million, with an average cost reduction of 10.4%. For 90-count prescriptions, 24 of 25 drugs (96%) demonstrated savings of $344 million, ranging from 2.0% to 99.3%.

**Table 1.** Potential combined savings (Medicare reimbursement and beneficiary out-of-pocket payments) if Medicare contracted rates matched MCCPDC’s pricing model.

Generic drug name	Total Medicare dosage units, 2022	Total Medicare spending, 2022	Annual MCCPDC cost, 30 count	Estimated annual savings, 30 count	Annual cost reduction %, 30 count	Estimated monthly savings, 30 count	Total MCCPDC cost, 90 count	Estimated annual savings, 90 count	Annual cost reduction %, 90 count	Estimated monthly savings, 90 count
Carbamazepine (generic for Tegretol)	134 300 647.46	$56 133 104.60	$39 842 525.41	−$16 290 579.19	−29.02%	−$1 357 548.27	$24 920 231.25	−$31 212 873.35	−55.61%	−$2 601 072.78
Carbamazepine (generic for Epitol)	1 113 352.50	$506 699.93	$252 359.90	−$254 340.03	−50.20%	−$21 195.00	$132 365.24	−$374 334.69	−73.88%	−$31 194.56
Celecoxib	289 072 167.02	$220 695 826.66	$85 758 076.22	−$134 937 750.44	−61.14%	−$11 244 812.54	$53 638 946.55	−$167 056 880.11	−75.70%	−$13 921 406.68
Diclofenac Potassium (generic for Zipsor)	113,772.00	$1,758,166.39	$292,014.80	−$1,466,151.59	−83.39%	−$122,179.30	$279,373.47	−$1,478,792.92	−84.11%	−$123,232.74
Diclofenac Potassium (generic for Lofena)	207,245.00	$6,394,727.91	$6,289,194.93	−$105,532.98	−1.65%	−$8,794.41	$6,266,167.71	−$128,560.20	−2.01%	−$10,713.35
Diclofenac Sodium ER	10,044,345.60	$14,838,756.27	$9,106,873.34	−$5,731,882.93	−38.63%	−$477,656.91	$7,990,834.94	−$6,847,921.33	−46.15%	−$570,660.11
Diclofenac-Misoprostol	3,610,412.00	$7,946,208.40	$2,515,253.69	−$5,430,954.71	−68.35%	−$452,579.56	$2,114,096.80	−$5,832,111.60	−73.39%	−$486,009.30
Diflunisal	752,758.50	$903,360.81	$727,666.55	−$175,694.26	−19.45%	−$14,641.19	$644,026.72	−$259,334.09	−28.71%	−$21,611.17
Etodolac	21,132,395.26	$14,702,324.84	$7,114,573.07	−$7,587,751.77	−51.61%	−$632,312.65	$4,766,529.15	−$9,935,795.69	−67.58%	−$827,982.97
Etodolac ER	1,569,128.00	$2,687,603.13	$1,312,837.09	−$1,374,766.04	−51.15%	−$114,563.84	$1,138,489.54	−$1,549,113.59	−57.64%	−$129,092.80
Fenoprofen Calcium	112,074.00	$329,135.93	$405,334.30	$76,198.37	23.15%	$6,349.86	$392,881.63	$63,745.70	19.37%	$5,312.14
Fludrocortisone Acetate	27,925,141.83	$12,635,559.10	$12,193,978.60	−$441,580.50	−3.49%	−$36,798.38	$9,091,185.06	−$3,544,374.04	−28.05%	−$295,364.50
Ibuprofen	440,199,381.12	$62,522,093.70	$90,974,538.76	$28,452,445.06	45.51%	$2,371,037.09	$42,063,496.42	−$20,458,597.28	−32.72%	−$1,704,883.11
Ibuprofen-Famotidine	1,045,391.00	$6,644,675.22	$864,189.89	−$5,780,485.33	−86.99%	−$481,707.11	$748,035.34	−$5,896,639.88	−88.74%	−$491,386.66
Indomethacin	15,422,680.00	$3,426,292.28	$4,575,395.07	$1,149,102.79	33.54%	$95,758.57	$2,861,763.96	−$564,528.32	−16.48%	−$47,044.03
Indomethacin Extended Release (ER)	15,422,680.00	$3,426,292.28	$4,112,714.67	$686,422.39	20.03%	$57,201.87	$2,399,083.56	−$1,027,208.72	−29.98%	−$85,600.73
Ketorolac Tromethamine	10,866,627.76	$36,982,531.36	$5,940,423.18	−$31,042,108.18	−83.94%	−$2,586,842.35	$4,733,020.09	−$32,249,511.27	−87.20%	−$2,687,459.27
Lidocaine	62,296,203.70	$166,928,674.23	$100,712,195.99	−$66,216,478.24	−39.67%	−$5,518,039.85	$93,790,395.58	−$73,138,278.65	−43.81%	−$6,094,856.55
Mefenamic Acid	18,507.00	$101,801.76	$13,448.42	−$88,353.34	−86.79%	−$7,362.78	$11,392.09	−$90,409.67	−88.81%	−$7,534.14
Meloxicam	19,482.00	$243,736.83	$3,831.46	−$239,905.37	−98.43%	−$19,992.11	$1,666.79	−$242,070.04	−99.32%	−$20,172.50
Nabumetone	48,918,258.83	$15,007,005.91	$15,001,599.37	−$5,406.54	−0.04%	−$450.54	$9,566,237.28	−$5,440,768.63	−36.25%	−$453,397.39
Naproxen	192,389,179.77	$41,842,966.54	$58,999,348.46	$17,156,381.92	41.00%	$1,429,698.49	$37,622,772.93	−$4,220,193.61	−10.09%	−$351,682.80
Naproxen Sodium ER	3,356,319.20	$3,258,596.36	$36,740,507.51	$33,481,911.15	1027.49%	$2,790,159.26	$36,367,583.15	$33,108,986.79	1016.05%	$2,759,082.23
Piroxicam	4,080,603.16	$5,881,327.50	$1,128,966.88	−$4,752,360.62	−80.80%	−$396,030.05	$675,566.52	−$5,205,760.98	−88.51%	−$433,813.41
Sulindac	13,391,660.00	$4,316,599.25	$4,508,525.53	$191,926.28	4.45%	$15,993.86	$3,020,563.31	−$1,296,035.94	−30.02%	−$108,002.99
**Totals cost and average % change**	1,297,380,412.71	$690,114,067.19	$489,386,373.10	−$200,727,694.09	10.42%	−$669,092.31	$345,236,705.08	−$344,877,362.11	−8.37%	−$1,149,591.21

Celecoxib exhibited the highest savings potential, totaling $134 million. Overall, savings for 30-count prescriptions ranged from $5406 to $134 million, with only 5 drugs showing savings below $200 000. For 90-count prescriptions, savings varied from $1666 to $93 million, averaging savings of $13 million. Not all medications yielded cost savings; Naproxen Sodium ER, for instance, showed the largest difference, with Medicare offering $33 million in savings for both 30- and 90-count prescriptions compared with MCCPDC rates.

## Discussion

MCCPDC’s cost-saving potential is evident: Celecoxib alone could have saved $134 million, contributing to the $344 million total savings across 25 medications. Celecoxib’s savings potential highlights the need to address pricing inefficiencies for prevalent chronic conditions like osteoarthritis and rheumatoid arthritis in Medicare beneficiaries.^6^

Our findings underscore inefficiencies in Part D pricing, recognizing that our savings calculations encompass total Medicare Part D expenditures, including both Medicare reimbursements and beneficiary out-of-pocket payments, rather than exclusively Medicare’s direct costs.

### Broader implications

Of the 25 medications, 19 (76%) demonstrated savings with 30-count prescriptions ($200 million total), while 24 (96%) had 90-count savings totaling $344 million, underscoring MCCPDC’s transformative pricing potential for high-demand medications.

The 2022 Inflation Reduction Act caps out-of-pocket costs and allows Medicare to negotiate prices for brand-name drugs but excludes generics (91% of prescriptions).^7^^,^^8^ A 2018 study suggests that transitioning from brand-name to generic medications could save Medicare $3 billion annually, reducing beneficiaries’ out-of-pocket expenses by $491 million per year.^9^ A 2021 study also showed that Medicare Part D could have saved $977 million in a single year by using generic equivalents.^10^ Despite these potential savings, generics remain underutilized. Our analysis relied on gross Medicare Part D spending (Medicare, plan, and patient payments) without accounting for confidential rebates, potentially leading to overestimates of net expenditures. Net-of-rebate administrative data are necessary to clarify actual Medicare savings. Nevertheless, our findings support further policy efforts to optimize US drug pricing. Importantly, our savings estimates reflect total Part D expenditures, not just Medicare’s portion, as CMS data include both reimbursements and out-of-pocket contributions.

### Limitations

This study used Medicare Part D data from 2022 and MCCPDC pricing from 2024, which might not reflect future pricing. We also did not account for other PBM-based discount models, such as GoodRx. Furthermore, we treated each “unit” as a single pill in accordance with the CMS Data Dictionary, whereas National Drug Codes consider a 30-day supply as one unit, and MCCPDC lists 28- or 30-day supply prices that we converted to per-pill costs, potentially introducing discrepancies in cost estimates. Finally, potential challenges in scaling MCCPDC pricing to Medicare were not explored. Future analyses incorporating manufacturer rebate data could isolate net Medicare spending from beneficiary out-of-pocket costs to more precisely estimate direct savings to Medicare.

## Data Availability

Our methodology and protocol in its entirety is publicly available on OSF to ensure transparency and reproducibility (https://osf.io/93g7f/). Data were released on November 29, 2024, and will remain infinitely available.
